# Meta-Analysis of Human Antibodies Against *Plasmodium falciparum* Variable Surface and Merozoite Stage Antigens

**DOI:** 10.3389/fimmu.2022.887219

**Published:** 2022-06-09

**Authors:** Eizo Takashima, Bernard N. Kanoi, Hikaru Nagaoka, Masayuki Morita, Ifra Hassan, Nirianne M. Q. Palacpac, Thomas G. Egwang, Toshihiro Horii, Jesse Gitaka, Takafumi Tsuboi

**Affiliations:** ^1^ Division of Malaria Research, Proteo-Science Center, Ehime University, Matsuyama, Japan; ^2^ Centre for Research in Infectious Diseases, Directorate of Research and Innovation, Mount Kenya University, Thika, Kenya; ^3^ Department of Malaria Vaccine Development, Research Institute for Microbial Diseases, Osaka University, Suita, Japan; ^4^ Med Biotech Laboratories, Kampala, Uganda; ^5^ Division of Cell-Free Sciences, Proteo-Science Center, Ehime University, Matsuyama, Japan

**Keywords:** *Plasmodium falciparum*, variable surface antigens, PfEMP1, RIFIN, malaria immunity, PCA

## Abstract

Concerted efforts to fight malaria have caused significant reductions in global malaria cases and mortality. Sustaining this will be critical to avoid rebound and outbreaks of seasonal malaria. Identifying predictive attributes that define clinical malaria will be key to guide development of second-generation tools to fight malaria. Broadly reactive antibodies against variable surface antigens that are expressed on the surface of infected erythrocytes and merozoites stage antigens are targets of naturally acquired immunity and prime candidates for anti-malaria therapeutics and vaccines. However, predicting the relationship between the antigen-specific antibodies and protection from clinical malaria remains unresolved. Here, we used new datasets and multiple approaches combined with re-analysis of our previous data to assess the multi-dimensional and complex relationship between antibody responses and clinical malaria outcomes. We observed 22 antigens (17 PfEMP1 domains, 3 RIFIN family members, merozoite surface protein 3 (PF3D7_1035400), and merozoites-associated armadillo repeats protein (PF3D7_1035900) that were selected across three different clinical malaria definitions (1,000/2,500/5,000 parasites/µl plus fever). In addition, Principal Components Analysis (PCA) indicated that the first three components (Dim1, Dim2 and Dim3 with eigenvalues of 306, 48, and 29, respectively) accounted for 66.1% of the total variations seen. Specifically, the Dim1, Dim2 and Dim3 explained 52.8%, 8.2% and 5% of variability, respectively. We further observed a significant relationship between the first component scores and age with antibodies to PfEMP1 domains being the key contributing variables. This is consistent with a recent proposal suggesting that there is an ordered acquisition of antibodies targeting PfEMP1 proteins. Thus, although limited, and further work on the significance of the selected antigens will be required, these approaches may provide insights for identification of drivers of naturally acquired protective immunity as well as guide development of additional tools for malaria elimination and eradication.

## Introduction

There is a common agreement that sustainable elimination of malaria will require multiple approaches (1). The need to develop efficacious vaccines remains an integral component of this fight, supported by strong evidence showing that naturally acquired protective immunity against *Plasmodium falciparum* is acquired among individuals living in malaria-endemic regions (2, 3). Specifically, antibody-mediated immunity against *P. falciparum* malaria is acquired with age and repeated exposure (2). This immunity mainly targets antigens of the parasite asexual blood-stages; however, the complete repertoire of the specific targets has not been unequivocally defined. Based on this understanding, several approaches are being applied to select the most appropriate vaccine candidates.

Focused and unbiased immuno-epidemiology studies have reported, identified, or characterized most of the leading candidate vaccines in the developmental pipeline. Most recently, high-throughput immunoscreening to simultaneously investigate proteins as potential vaccine candidates or as immune correlates of protection has been a major strategy (4–7) with antigens such as PfRh5, CelTOS, MSP3, and GLURP (7–9) being identified. These antigens are in the vaccine development pipeline. However, most data, to date has limited our ability to prioritize antigens, enrich the pool of vaccine candidates, and link immunological data with clinical outcomes.

Over the years, we have developed and optimized a robust and high-throughput eukaryotic wheat germ cell-free protein synthesis system (WGCFS) coupled with a homogeneous high-throughput AlphaScreen platform for antibody profiling and mechanistic characterization of proteins that have a role in merozoites invasion of erythrocytes, or induction of protective immunity in malaria naturally exposed individuals (9–11). Leveraging this approach, we have prioritized, from hundreds of parasite proteins expressed in multiple parasite stages, several *P. falciparum* antigens for inclusion in the vaccine development pipeline. Recently, a total of 1,827 recombinant proteins drawn from different *P. falciparum* stages (sporozoites, merozoites, trophozoites, schizonts, and gametocytes) were used to probe individual serum samples obtained from residents of a malaria endemic region in Uganda. Protein immunoreactivity was observed at 54% with 128 antigens inducing antibody responses that significantly associated with reduced risk to clinical malaria episodes (defined as fever ≥37.5 ˚C and asexual parasitemia of ≥2,500/µl of blood) during a 12-months follow-up period. Of these antigens, 53 were down-selected as the most viable vaccine candidates by virtue of having a signal peptide (SP) and/or transmembrane domain (TM) (4, 5, 7) suggesting their putative expression on the surface of merozoites and/or sporozoites, or on the infected erythrocytes. Similarly, by focusing on parasite protein families that are exported to the surface of infected red blood cells such as erythrocyte membrane protein 1 (PfEMP1), repetitive interspersed family (RIFIN) proteins, subtelomeric variable open reading frame (STEVOR), and surface-associated interspersed gene family (SURFIN) (12), we observed that more than 95% of the antigens were reactive with serum samples obtained from Uganda (11, 13). These studies demonstrated that the repertoire of potentially protective antigens that correlated with protective immunity against clinical malaria is wider than thought and offers multiple options for the identification of malaria vaccine candidates, aside from those that are currently under clinical or pre-clinical evaluation (9).

Previous studies with our follow-up cohort in Uganda (14) and similar immunoepidemiology studies (4, 7, 15) have largely focused on a single definition of clinical malaria based on the incidence within a specified geographic area. However, ongoing field trials/studies further strengthen the argument that clinical definitions of malaria are also influenced by factors that are related to host immunity (age, transmission, co-infections, etc), and parasitaemia accompanied by symptoms do not necessarily imply clinical malaria especially in endemic areas (16–19). Moreover, malaria transmission heterogeneity (ranging from high, stable, and declining), as well as absence of robust correlates of protection highlights the complexity involved in evaluating new and future interventions. In this study, to obtain a wider picture, and considering the declining malaria transmission in endemic areas, we sought to re-evaluate the previously published data together with new data sets generated against merozoites stage antigens that are considered potential targets of protective immunity, to assess the relationship between antibody responses and clinical malaria outcomes categorized by different clinical malaria definitions. Further, using the Principal Component Analysis (PCA) for the antibody responses we highlight antigens that are robustly identified by different protein based global immunological screens.

## Materials and Methods

### Study Setting and Ethical Statement

Serum samples used in this study were obtained from residents of Lira Municipality, Northern Uganda, that were taking part in a prospective study of 66 non-vaccinated participants aged 6–20 years. The region was characterized by high malaria transmission (14, 20–23). Study protocols and permission to use the samples were approved by the Institutional Research and Ethics Committee of Lacor Hospital (LHIREC 023/09/13), Uganda National Council for Science and Technology (HS1403) in Uganda; and Ethics Committees of the Research Institute for Microbial Diseases, Osaka University, and Ehime University, Japan. Written informed consents were obtained from all participants and/or their parents or guardians before the study. Aside from parental consent, assent was obtained from children aged 8-17 years. The study was conducted in compliance with the International Conference on Harmonisation Good Clinical Practices guidelines and the Declaration of Helsinki.

### Production of a *P. falciparum* Parasite Protein Library

The comprehensive protein library consisted of 579 proteins representing the asexual erythrocytic stages of *P. falciparum* (**Table S1**). Newly synthesized asexual blood-stage proteins (BSP: n = 46) selected from our previous study based on their significant immunoreactivities (9) were asssessd together with data from previously published libraries of proteins derived from cysteine-rich interdomain regions of *P. falciparum* erythrocyte membrane protein 1 (PfEMP1) (CIDR: n = 108), Duffy binding–like domains of PfEMP1 (DBL: n = 163) (11), repetitive interspersed family proteins (RIFIN: n = 176), subtelomeric variable open reading frame proteins (STEVOR: n = 53), and surface-associated interspersed gene family proteins (SURFIN: n = 33) (13). All proteins were synthesized using the optimized WGCFS protocol and assayed alongside biotinylated rabbit IgG standard for plate-to-plate and day-to-day normalization (11).

All the proteins were expressed from sequences derived from the *P. falciparum* 3D7 reference strain. Briefly, the DNA sequences representing the ectodomains while excluding the SP and/or TM domains were amplified by using high fidelity PrimeSTAR Max DNA polymerase (Takara Bio, Kusatsu, Japan) and cloned into the WGCFS dedicated pEU plasmid vector (CellFree Sciences, Matsuyama, Japan) using the In-Fusion HD Cloning Kit (Takara Bio). A semi-automated GenDecoder1000 robotic protein synthesizer (CellFree Sciences) was used for *in vitro* transcription and mono-biotinylated recombinant protein synthesis with WGCFS.

### Human Antibodies Quantification by AlphaScreen

We used the AlphaScreen platform to assess serum antibodies in malaria exposed individuals as described (9, 24). The system exploits the existence of mono-biotinylation on each recombinant *P. falciparum* protein. Specifically, the proteins were dispensed into a 384-well OptiPlate using a JANUS Automated Workstation dispenser (PerkinElmer, Waltham, MA) and mixed with 10 μl of 4000-fold diluted sera in reaction buffer (100 mM Tris-HCl [pH 8.0], 0.01% [v/v] Tween-20, and 0.1% [w/v] bovine serum albumin). After 30 min incubation at 26°C, 10 μl of detection mixture containing streptavidin-coated donor beads (PerkinElmer) and protein G (Thermo Scientific, Waltham, MA) conjugated acceptor beads (PerkinElmer) were added, to make a final concentration of 12 μg/ml for both beads. The plate was then incubated in the dark for 1 hr at 26°C to allow optimal binding of the donor and acceptor beads to the biotinylated protein and human antibody, respectively. Luminescence emitted by acceptor beads upon excitation of the donor beads was detected using an EnVision plate reader (PerkinElmer) and captured as AlphaScreen Counts (ASC). To account for day-to-day and plate-to-plate assay variability, serially diluted biotinylated rabbit IgG (PerkinElmer) was included in each plate, and subsequently used to generate a 5-parameter logistic standardization curve. The assays were randomized to minimize experimental bias.

### Statistical Analysis

All data analyses were performed using R software (Version 4.0.1, R Foundation for Statistical Computing). As previously described, protein seropositivity cut off point to human sera was set at half the lowest non-negative ASC value from that of the assayed samples (25), and a protein was considered immunoreactive if more than 10% of the volunteers had ASC levels above the seropositivity cut-off.

For survival analysis, the time-to-first clinical malaria episode from baseline (defined as the time from first sampling when all enrolled individuals were blood-smear negative) was used as the endpoint (14). For detailed analyses, three different definitions for clinical malaria were used: fever ≥37.5°C and asexual parasitemia of (i) ≥1,000/μl, (ii) ≥2,500/μl, and (iii) ≥5,000/μl blood, with no sign(s) of complicated disease. Although some children may have multiple parasitic or febrile malaria episodes as defined above, only the first febrile episode was considered in the analysis. To assess whether the presence of antigen-specific antibodies was associated with overall survival, Cox proportional hazards model was used to calculate hazard ratios (HR) and 95% confidence intervals (CIs) between ‘High Responders’ (individuals with an ASC value above the population median for that antigen) or ‘Low Responders’ (7, 9, 26, 27). Potential protective efficacy (PPE) relative to different definitions of malaria was computed as 1- hazard ratio (PPE% = (1- HR) × 100%). For multivariate survival analysis, data was adjusted for bednet use and age as a categorical variable (6-10 years, 11-15 years, and 16-20 years).

To simultaneously evaluate antibodies developed in response to different proteins among patients with and without clinical malaria, we used Principal Component Analysis (PCA). PCA essentially reduces the dimensionality of antibody responses by generating fewer composite variables to capture as much variance in that dataset. The model then assesses and compares the relationship between the scores obtained for each of these components per subject. To generate the global antigen derived principal components, all immunoreactive antigens were used without prespecified groupings (BSP, PfEMP1-CIDR, PfEMP1-DBL, RIFIN, STEVOR, and SURFIN) to determine group-based variance. To identify PCA derived clusters of antibody responses that were involved in protection against clinical malaria, we selected principal components where at least three variables were loaded, and the eigenvalue was greater than 2 or the proportion of variance explained was >5% (28). Individuals displaying outlier PCA coordinates were excluded. Individual contributions to the PCA were assessed in association with age, gender, and clinical malaria outcome.

## Results

### Seroprevalence of Antibodies to Different *P. falciparum* Proteins

**Figure 1:**
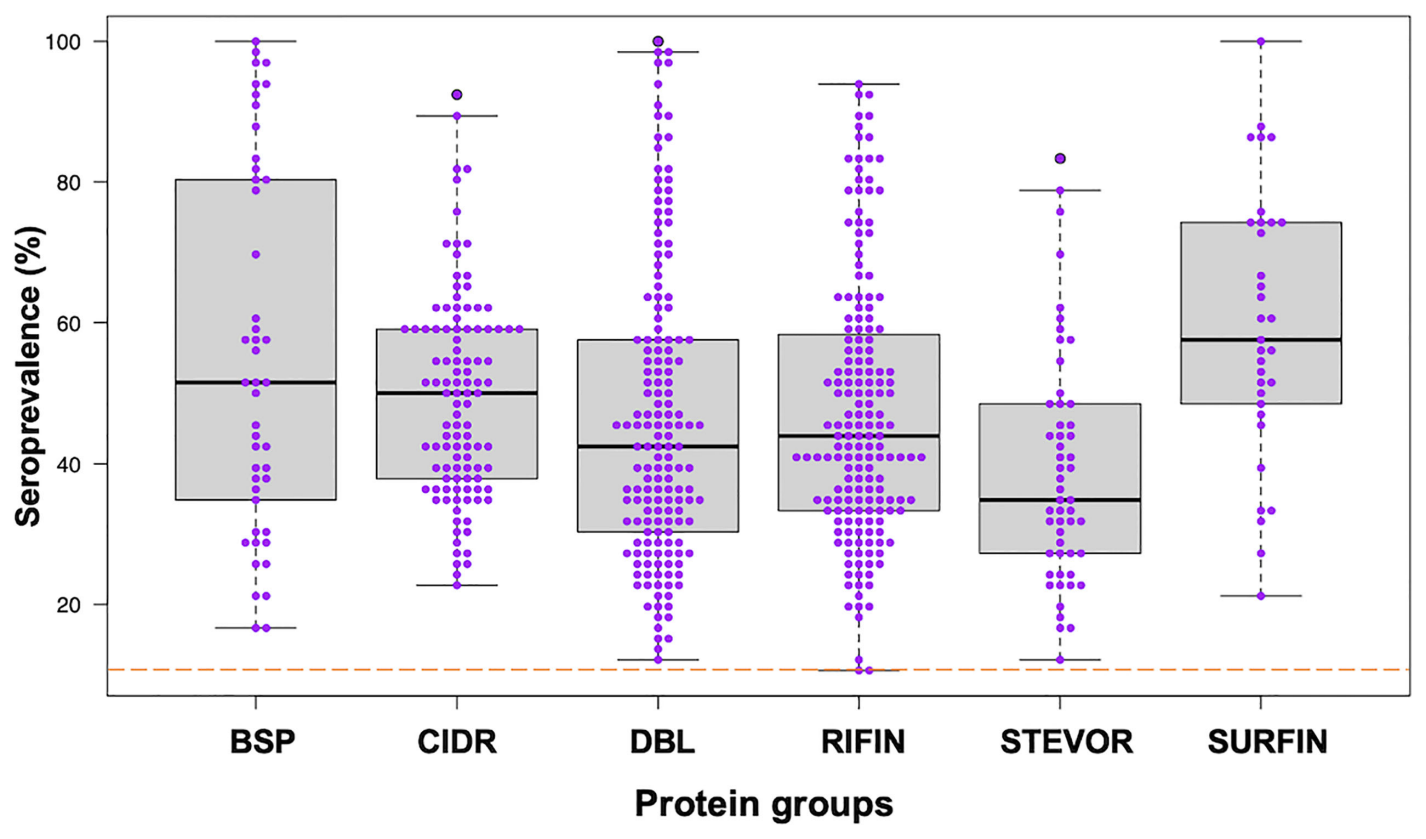
Seroprevalence of antibodies to different *P. falciparum* proteins. Antibodies immunoreactive to BSP (blood-stage proteins), CIDR (cysteine-rich interdomain regions of PfEMP1), DBL (Duffy binding–like domains of PfEMP1), RIFIN (repetitive interspersed family proteins), STEVOR (subtelomeric variable open reading frame proteins), and SURFIN (surface-associated interspersed gene family proteins) are shown. Box plots illustrate medians with 25^th^ and 75^th^ and whiskers for 10^th^ and 90^th^ percentiles with the horizontal line in each group denoting the overall median per group. The dashed red horizontal line indicates 10% seroprevalence that was set as protein immunoreactivity cut-off point. The data in BSP were newly measured in this study. The data in other protein groups, CIDR and DBL from PfEMP1, RIFIN, STEVOR and SURFIN were reported previously (11, 13).

We assessed the human antibody reactivities to the protein library (**Table S1**) obtained from the samples (n = 66) taken at the beginning of the study, prior to the rainy season. All the blood-stage proteins (BSP) were newly measured in this study and they were all immunoreactive with seroprevalence above the threshold cut-off point of 10%. Seroprevalence of the other protein groups was reported previously (11, 13). Briefly, 99% immunoreactivity was observed for PfEMP1 (CIDR and DBL), RIFIN, STEVORs, and SURFINs (11, 13) (**Figure 1**). The seroprevalence varied widely among the protein groups/families: BSP (16-100%), CIDR domains (22-92%), DBL domains (12-100%), RIFIN (10-93%), STEVORs (12-83%) and SURFINs (21-100%) (Kruskal-Wallis Test, *p* = 0.001) with STEVORs having significantly lower median levels, and SURFINs the highest compared to other groups. Although this may represent cross-reactivity within the assessed families (12), a strong Spearman’s rank correlation between antibody levels measured in the samples and across different groups, as shown by the correlation matrix (**Figure S1** **)** suggests that antibodies to different antigens are co-acquired in this population.

### Relationship Between Antibodies and Incidence of Febrile Malaria

**Figure 2:**
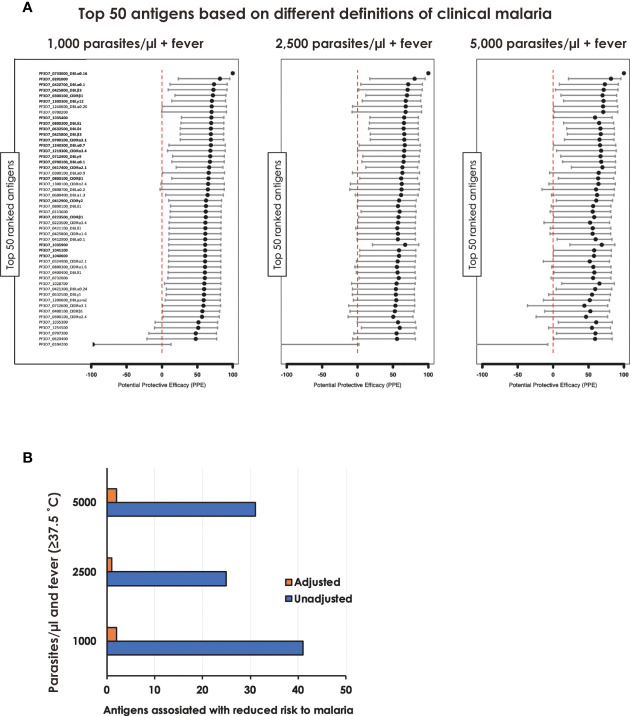
Associations between antibody levels and risk of *P. falciparum* febrile malaria. Complete list of the data for potential protective efficacy (PPE), and 95% confidence interval, is provided in **Table 1**. **(A)** Unadjusted association between antibody levels and risk of *P. falciparum* febrile malaria for each of the antigens tested in the cohort. The top-50 antigens are ranked - top to bottom - by the strength of their PPE. PPE for each antigen was derived from the hazard ratio (HR) calculated by the unadjusted Cox-regression hazard model analysis (comparing children with high vs. low antibody responses). Black dots indicate the percentage protection, and error bars indicate the 95% confidence interval. The red vertical line represents a PPE of 0% (i.e. HR = 1). The 22 cross-selected antigens are labeled in bold. **(B)** The bars represent the number of antigens that associated with protection to clinical malaria in univariate and multivariate analysis. Red bar: Adjusted, Blue bar: Unadjusted.

To gain further insight into the relationship between individual protein reactivity and clinical malaria outcomes, we determined the hazard ratios based on time-to-event using three parasite thresholds for clinical malaria in univariate analysis. Based on the lower threshold of 1,000 parasites/µl blood, antigen-specific antibodies to 43 proteins associated with protection. This changed to 26 and 32 proteins when assessed at 2,500 parasites/µl blood and a higher threshold of 5,000 parasites/µl blood, respectively (**Figure 2A**). In the multivariable-adjusted survival analysis, the correlation between antibodies and the risk of febrile malaria was generally reduced. Twenty-two antigens were selected across the three definitions (**Table 1**) by the unadjusted analysis. The 22 antigens included 17 PfEMP1 domains, 3 RIFIN family members, merozoite surface protein 3 (PF3D7_1035400), and merozoite-associated armadillo repeats protein (PF3D7_1035900). The number of antigens that remained significantly associated with reduced risk of clinical malaria after adjusting for age (24) and bed-net use was 2 for 1,000 parasites/µl blood, 1 for 2,500 parasites/µl blood, and 2 antigens for 5,000 parasites/µl blood (summarized in **Figure 2B**).

**Table 1:** Associations between antibody levels and risk of *P. falciparum* clinical malaria.

		Unadjusted PPE (1,000 parasites/µl)				Unadjusted PPE (2,500 parasites/µl)				Unadjusted PPE (5,000 parasites/µl)			
Product ID	GeneID*	PPE %	Lower 95%CI	Upper 95%CI	P value**	PPE %	Lower 95%CI	Upper 95%CI	P value**	PPE %	Lower 95%CI	Upper 95%CI	P value**
DC150	**PF3D7_0733000_DBLα0.16**	1.000	0.990	1.000	0.001	1.000	0.990	1.000	0.001	1.000	0.990	1.000	0.001
RS23	**PF3D7_0201000**	0.819	0.230	0.957	0.021	0.806	0.174	0.954	0.027	0.817	0.218	0.957	0.022
DC54	**PF3D7_0420700_DBLα0.1**	0.735	0.114	0.921	0.031	0.716	0.046	0.915	0.042	0.730	0.089	0.920	0.035
DC72	**PF3D7_0425800_DBLβ3**	0.727	0.087	0.918	0.035	0.707	0.017	0.913	0.047	0.714	0.035	0.915	0.044
DC21	**PF3D7_0300100_CIDRβ1**	0.720	0.184	0.904	0.020	0.698	0.117	0.897	0.029	0.698	0.111	0.898	0.030
DC266	**PF3D7_1300300_DBLγ12**	0.705	0.139	0.899	0.026	0.681	0.066	0.891	0.037	0.711	0.148	0.902	0.024
DC251	PF3D7_1240600_DBLα0.20	0.702	0.004	0.911	0.049	0.680	(0.072)	0.905	0.065	0.705	0.005	0.913	0.049
RS89	PF3D7_0700200	0.700	(0.002)	0.910	0.050	0.679	(0.076)	0.904	0.066	0.708	0.015	0.914	0.047
5400	**PF3D7_1035400**	0.698	0.275	0.874	0.007	0.658	0.175	0.859	0.017	0.592	0.009	0.832	0.048
DC169	**PF3D7_0800300_DBLδ1**	0.695	0.268	0.873	0.008	0.655	0.167	0.857	0.018	0.650	0.148	0.856	0.021
DC110	**PF3D7_0632500_DBLδ4**	0.691	0.259	0.871	0.008	0.651	0.157	0.855	0.019	0.668	0.191	0.864	0.015
DC73	**PF3D7_0425800_DBLβ3**	0.691	0.259	0.871	0.009	0.660	0.179	0.859	0.016	0.672	0.201	0.866	0.014
DC119	**PF3D7_0700100_CIDRα3.1**	0.691	0.259	0.871	0.009	0.660	0.179	0.859	0.016	0.655	0.160	0.858	0.019
DC242	**PF3D7_1240300_DBLα0.7**	0.691	0.098	0.894	0.032	0.667	0.024	0.886	0.045	0.663	0.007	0.885	0.049
DC239	**PF3D7_1219300_CIDRα3.4**	0.684	0.078	0.891	0.035	0.659	0.001	0.883	0.050	0.678	0.051	0.890	0.040
DC137	**PF3D7_0712400_DBLγ9**	0.681	0.151	0.881	0.022	0.655	0.074	0.871	0.035	0.669	0.108	0.878	0.029
DC118	**PF3D7_0700100_DBLα0.1**	0.677	0.139	0.879	0.024	0.650	0.061	0.869	0.037	0.679	0.135	0.881	0.025
DC102	**PF3D7_0617400_CIDRα2.1**	0.666	0.200	0.861	0.014	0.634	0.115	0.848	0.026	0.696	0.259	0.875	0.009
DC18	PF3D7_0300100_DBLα0.9	0.660	0.008	0.883	0.048	0.633	(0.073)	0.875	0.067	0.643	(0.050)	0.879	0.061
DC158	**PF3D7_0800100_CIDRβ1**	0.657	0.140	0.863	0.023	0.616	0.032	0.848	0.043	0.636	0.073	0.857	0.034
DC260	PF3D7_1300100_CIDRα2.4	0.652	(0.014)	0.881	0.053	0.624	(0.100)	0.872	0.074	0.641	(0.059)	0.878	0.063
DC175	PF3D7_0808700_DBLα0.2	0.648	(0.027)	0.879	0.056	0.621	(0.110)	0.870	0.077	0.607	(0.158)	0.866	0.090
DC99	PF3D7_0600400_DBLα1.3	0.644	0.051	0.867	0.039	0.615	(0.032)	0.856	0.058	0.621	(0.024)	0.860	0.056
DC49	**PF3D7_0412900_CIDRγ2**	0.622	0.094	0.842	0.029	0.586	0.001	0.829	0.050	0.609	0.045	0.840	0.039
DC157	PF3D7_0800100_DBLδ1	0.621	0.121	0.837	0.024	0.571	(0.004)	0.816	0.051	0.565	(0.028)	0.816	0.058
RS264	PF3D7_0113600	0.619	0.115	0.836	0.025	0.594	0.049	0.826	0.038	0.559	(0.041)	0.813	0.062
DC17	**PF3D7_0223500_CIDRβ1**	0.618	0.114	0.835	0.025	0.579	0.015	0.820	0.046	0.580	0.008	0.822	0.048
DC15	PF3D7_0223500_CIDRα3.4	0.613	0.102	0.833	0.027	0.573	0.002	0.817	0.050	0.522	(0.129)	0.798	0.092
DC64	PF3D7_0421100_DBLδ1	0.613	0.102	0.833	0.027	0.561	(0.026)	0.812	0.057	0.559	(0.040)	0.813	0.061
DC71	PF3D7_0425800_CIDRα1.6	0.612	0.101	0.833	0.027	0.571	(0.004)	0.816	0.051	0.558	(0.044)	0.813	0.063
DC46	PF3D7_0412900_DBLα0.1	0.611	0.097	0.832	0.028	0.569	(0.007)	0.816	0.052	0.601	0.057	0.831	0.036
WE35	**PF3D7_1035900**	0.610	0.095	0.832	0.028	0.673	0.209	0.864	0.013	0.687	0.236	0.872	0.011
RS163	**PF3D7_1041100**	0.610	0.095	0.832	0.028	0.585	0.031	0.823	0.042	0.579	0.007	0.822	0.048
RS158	**PF3D7_1040600**	0.610	0.095	0.832	0.028	0.585	0.031	0.823	0.042	0.577	0.001	0.821	0.050
DC23	PF3D7_0324900_CIDRα2.1	0.609	0.093	0.831	0.029	0.567	(0.013)	0.815	0.053	0.517	(0.139)	0.795	0.097
DC166	PF3D7_0800300_CIDRα1.6	0.608	0.092	0.831	0.029	0.556	(0.038)	0.810	0.061	0.568	(0.021)	0.817	0.056
DC36	PF3D7_0400400_DBLδ1	0.606	0.086	0.830	0.030	0.565	(0.018)	0.814	0.055	0.582	0.013	0.823	0.047
RS99	PF3D7_0732000	0.605	0.084	0.830	0.030	0.581	0.021	0.821	0.045	0.564	(0.029)	0.816	0.058
WE29	PF3D7_1028700	0.598	0.036	0.832	0.041	0.551	(0.085)	0.814	0.075	0.654	0.122	0.864	0.026
DC66	PF3D7_0421300_DBLα0.24	0.595	0.062	0.826	0.035	0.542	(0.072)	0.804	0.072	0.595	0.043	0.828	0.039
DC109	PF3D7_0632500_DBLγ1	0.594	0.059	0.825	0.036	0.541	(0.074)	0.804	0.073	0.550	(0.064)	0.809	0.069
DC232	PF3D7_1200600_DBLpam2	0.589	0.047	0.823	0.038	0.547	(0.058)	0.806	0.067	0.518	(0.137)	0.796	0.096
DC139	PF3D7_0712600_CIDRα3.1	0.586	0.007	0.827	0.048	0.534	(0.124)	0.807	0.089	0.441	(0.362)	0.771	0.200
DC30	PF3D7_0400100_CIDRβ1	0.571	0.005	0.815	0.049	0.527	(0.106)	0.798	0.084	0.525	(0.120)	0.799	0.089
DC19	PF3D7_0300100_CIDRα2.4	0.566	0.016	0.808	0.046	0.504	(0.134)	0.783	0.096	0.465	(0.240)	0.769	0.145
WE50.1	PF3D7_1035300	0.515	(0.097)	0.786	0.082	0.571	(0.004)	0.817	0.051	0.608	0.073	0.834	0.033
RS189	PF3D7_1254500	0.512	(0.105)	0.784	0.085	0.596	0.055	0.827	0.037	0.549	(0.065)	0.809	0.069
WE48	PF3D7_0707300	0.477	(0.185)	0.769	0.120	0.551	(0.051)	0.808	0.065	0.598	0.048	0.830	0.038
MSP10_1-1	PF3D7_0620400	0.477	(0.214)	0.774	0.131	0.558	(0.068)	0.817	0.070	0.593	0.009	0.833	0.048
WE27	PF3D7_0104200	(0.977)	(3.478)	0.127	0.102	(1.293)	(4.364)	0.019	0.056	(1.618)	(5.368)	(0.076)	0.034

*In bold are the proteins cross-selected by the three malaria definations.

**Yellow highlighted P values: statistically significant by the unadjusted analysis.

### Principal Component Analysis of the Antibody Responses

**Figure 3:**
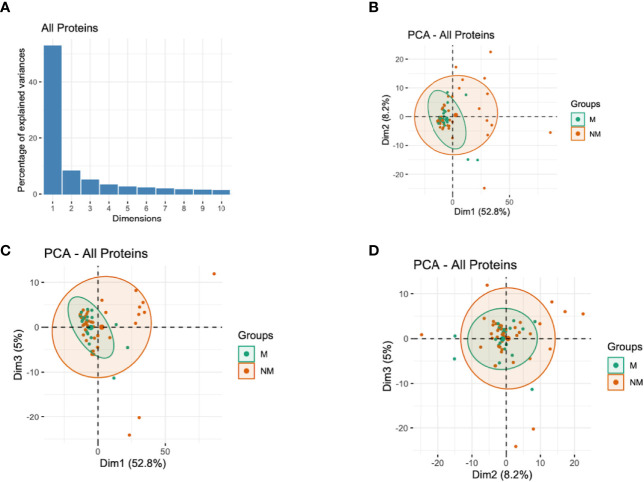
Principal Components Analysis (PCA) of the antibody responses. **(A)** Distribution of principal components (first 10 dimensions) that explained the highest variance of the antibody responses derived from all the immunoreactive proteins. **(B–D)** The plots of the distribution of individuals by type of antibody responses to different proteins. The principal components (Dim)1 vs 2 **(B)**, Dim1 vs 3 **(C)**, and Dim2 vs 3 **(D)** that explained the highest percentage of the variance (percentage in parenthesis) of the antibody responses are presented. Green and red represent malaria (M) and no malaria (NM) cases, respectively. Light green and light red eclipses represent distribution of individuals with and without clinical malaria episodes, respectively.

Since the antibody responses and the corresponding semi-protective immunity involve numerous antibodies acting simultaneously but targeting different proteins (26, 29), we performed PCA with all the proteins assayed and with different protein families to capture the effects of all antibodies in a single analysis and extract important associations. The analysis indicated that the first three components (with eigenvalues of 306, 48, and 29, respectively) accounted for 66.1% of the total variation in these data (**Figure 3A**). The analysis showed that the participants who did not experience clinical malaria were more dispersed while those with malaria episodes clustered together (**Figures 3B–D**). The first component (Dim1) explained 52.8% of variability and gave the greatest weights to antibodies against PfEMP1 domains [PF3D7_1240300_DBLβ8 (contributing 0.3), PF3D7_0632500_DBLe2 (0.3), PF3D7_0200100_CIDRα2.2 (0.29), PF3D7_1000300 (0.29) and PF3D7_1373500_CIDRβ6 (0.28)] (**Table 2**; **Figure S2**), while principal component 2 (Dim2) was mainly reflective of anti-PF3D7_1372800 (0.78), PF3D7_0401400 (0.77), PF3D7_0632100 (0.74), PF3D7_0500400 (0.72), and PF3D7_0425900 (0.67) and accounted for 8.2% of the variation. The third component (Dim3), which explained slightly above 5.0% of data variability, gave the strongest weight to antibodies against PF3D7_1200100_DBLα0.9 (2.44), PF3D7_1150400_CIDRγ2 (2.33), PF3D7_0712300_CIDRα0.1 (2.21), PF3D7_0400400_DBLδ1 (2.07), and PF3D7_1200400_DBLγ14 (1.86) (**Table 2**; **Figure S2**). These findings suggest that PfEMP1 domains are major determinants of the variability observed in individuals experiencing clinical malaria and are consistent with the findings of the Cox analysis model (**Figure 2A**). Specifically, the PF3D7_0425800_DBLβ3 was the only domain selected by the multi-approach analysis and appeared as well in the top 50 antigens by PCA.

**Table 2:** Top 50 antigens based on respective contributions to variability in principal components 1, 2 and 3 (Dim1, Dim2, Dim3).

Product ID	GeneID	Dim1*	Dim2*	Dim3*
DC244	PF3D7_1240300_DBLβ8	**0.299**	0.033	0.014
DC113	PF3D7_0632500_DBLe2	**0.298**	0.022	0.014
DC12	PF3D7_0200100_CIDRα2.2	**0.293**	0.091	0.056
RS146	PF3D7_1000300	**0.287**	0.030	0.044
DC273	PF3D7_1373500_CIDRβ6	**0.286**	0.100	0.102
RS68	PF3D7_0425900	0.159	**0.673**	0.006
RS69	PF3D7_0500400	0.145	**0.722**	0.063
RS83	PF3D7_0632100	0.044	**0.739**	0.135
RS206	PF3D7_1372800	0.014	**0.778**	0.132
RS60	PF3D7_0401400	0.013	**0.769**	0.040
DC228	PF3D7_1200400_DBLγ14	0.097	0.039	**1.864**
DC36	PF3D7_0400400_DBLδ1	0.044	0.154	**2.074**
DC131	PF3D7_0712300_CIDRα0.1	0.043	0.028	**2.209**
DC220	PF3D7_1150400_CIDRγ2	0.037	0.049	**2.331**
DC221	PF3D7_1200100_DBLα0.9	0.028	0.112	**2.443**
DC231	PF3D7_1200600_DBLpam1	0.286	0.160	0.011
DC5	PF3D7_0100300_DBLα1.3	0.285	0.031	0.001
DC237	PF3D7_1200600_DBLe10	0.282	0.071	0.009
DC75	PF3D7_0425800_DBLδ1	0.281	0.076	0.058
DC25	PF3D7_0324900_CIDRβ7	0.281	0.183	0.036
DC180	PF3D7_0809100_CIDRα2.2	0.280	0.136	0.064
DC13	PF3D7_0200100_DBLγ11	0.278	0.185	0.000
RS184	PF3D7_1254100	0.278	0.049	0.050
RS204	PF3D7_1372600	0.278	0.107	0.029
RS63	PF3D7_0413200	0.278	0.053	0.021
DC78	PF3D7_0426000_CIDRα4	0.277	0.234	0.041
DC4	PF3D7_0100100_CIDRβ1	0.275	0.151	0.018
DC171	PF3D7_0808600_DBLα0.15	0.275	0.240	0.062
DC73	PF3D7_0425800_DBLβ3	0.275	0.034	0.017
RS185	PF3D7_1254200	0.274	0.030	0.042
DC224	PF3D7_1200100_CIDRβ1	0.221	0.016	0.676
DC132	PF3D7_0712300_DBLδ1	0.199	0.001	0.626
DC127	PF3D7_0712000_CIDRα3.1	0.195	0.622	0.078
DC143	PF3D7_0712800_CIDRα2.4	0.193	0.555	0.205
DC31	PF3D7_0400400_DBLα1.2	0.185	0.048	0.613
DC130	PF3D7_0712300_DBLα0.1	0.183	0.012	1.129
RS67	PF3D7_0425700	0.171	0.580	0.098
DC147	PF3D7_0712900_CIDRα3.1	0.164	0.638	0.154
DC149	PF3D7_0712900_CIDRβ1	0.163	0.619	0.115
DC213	PF3D7_1100200_CIDRβ4	0.152	0.535	0.005
DC35	PF3D7_0400400_DBLγ11	0.152	0.000	1.322
DC236	PF3D7_1200600_DBLepam5	0.142	0.127	0.980
DC152	PF3D7_0733000_DBLδ1	0.140	0.648	0.226
DC126	PF3D7_0712000_DBLα0.17	0.133	0.011	1.412
DC46	PF3D7_0412900_DBLα0.1	0.129	0.009	1.524
RS110	PF3D7_0800500	0.128	0.642	0.001
DC234	PF3D7_1200600_DBLpam3	0.125	0.031	1.079
RS101	PF3D7_0732200	0.119	0.001	0.758
DC232	PF3D7_1200600_DBLpam2	0.099	0.000	1.112

*In bold are the proteins with the highest contribution to principle component.

When assessing the representation of antibody responses to different protein groups based on Dim1 and Dim2, we observed that most clinical malaria cases clustered together irrespective of the protein group or family (**Figure S3**); and both components are the major drivers of the observed variability. This was again in agreement with the observation in Cox analysis which suggested that multiple, and probably specific antigens may be associated with protective immunity (**Figure 2A**).

**Figure 4:**
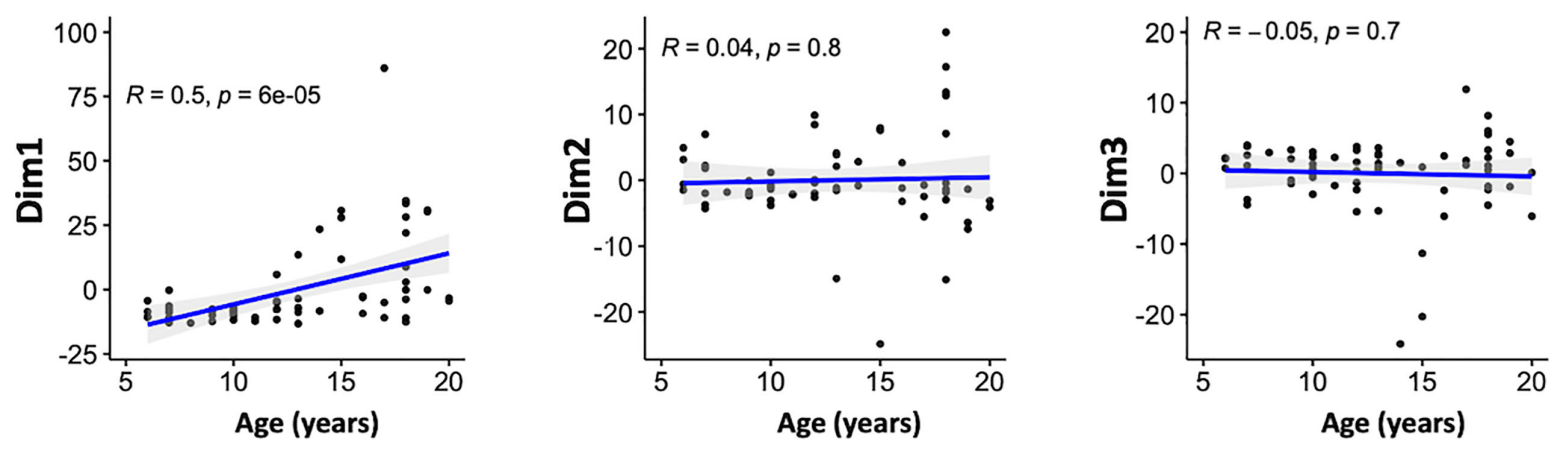
Associations between the antibody responses and age. Correlation between the scores of the principal components 1–3 and age (in years) of the participants. Blue line represent linear regression lines and shading represents 95% confidence intervals. Only the first principal component (Dim1) correlated with age.

We then assessed the relationship between the scores obtained from PCA on one hand and age and malaria clinical outcome to identify patterns of specific antibody responses that could be important in malaria. Age was significantly positively correlated with the scores of the first principal component (Dim1, *R* = 0.5; *P* <0.001) but not with Dim2 (*R* = 0.04, *P* = 0.8) or Dim3 (*R* = -0.05, *P* = 0.7) (**Figure 4**). This, further strengthens the observation on previous studies that age is an important factor on naturally acquired immunity (2).

## Discussion

Here, using antibody response data from our previous publications on multi-gene families (9, 11, 13) plus newly generated data from merozoite surface antigens, and expanding the analysis to include wider definition of clinical malaria (categorized according to different parasitemia thresholds), we re-assessed antibody responses in a malaria exposed population in Northern Uganda to capture as much immunological information as possible within the context of protective immunity from one population. This comprehensive approach would help identify further key antigenic targets or signature(s) of protective immunity. We observed 22 antigens, that included 17 PfEMP1 domains, 3 RIFIN family members, merozoite surface protein 3 (MSP3; PF3D7_1035400), and merozoite-associated armadillo repeats protein (PfMAAP; PF3D7_1035900), selected by the three clinical malaria definitions (1,000/2,500/5,000 parasites/µl blood plus fever). This suggest that these proteins have a key role in protection against clinical malaria.

We observed strong immune responses with high seroprevalence in the different protein families. This could be due to high level of exposure to these abundantly expressed proteins or cross-reactivity among the conserved regions of the domains (12). Although it could be argued that, likely, only highly expressed proteins can be flagged down in terms of antigen discovery and could be restrictive, the potential cross-reactivity could also be a strong point in terms of cross-protection. For instance, recent data suggests that despite their huge sequence diversity, CIDRα1 are structurally and functionally conserved for binding to EPCR (30, 31). Importantly, CIDRα1-EPCR interaction can be blocked by antibodies obtained from individuals naturally infected with malaria in Tanzania (30). The function of other multi-gene families in malaria infection and pathogenicity, to date, remains unclear but it is expected that the parasite could be using these proteins as alternative ligands to evade human immune system for host colonization.

Among them, PF3D7_0425800_DBLβ3, a PfEMP1 domain was selected by the multi-definition analyses, and appeared as well in the top 50 antigens by PCA. The molecule is a domain cassette 4 (DC4) family member consisting of a combination of short tandems that is associated with severe malaria (32–34). Antibodies against DBLβ3 can broadly inhibit PfEMP1 binding to intercellular adhesion molecule 1 (ICAM1) and are cross-reactive to DC4 derived from genetically distant parasite isolates (35). DBLβ3 was also identified in the previous analyses (32, 34) and the approach used here affirmed the importance of this domain in protection against symptomatic malaria. In addition, 3 members of the RIFIN family were selected. This is consistent with recent data which pointed to an important role of these molecules as ligands for opsonization of infected RBCs (36). Studies have suggested that anti-RIFIN antibodies correlate with parasite clearance, abrogation of symptoms of clinical malaria in children (37), and have a role in protection against severe malaria in Tanzanian patients (38). However, we do note that targeting RIFIN alone, as with PfEMP1 domains, for vaccine/as possible intervention tools may not be sufficient or highly effective due to the clonal expression of these proteins (39). We hypothesize that cross-conserved domains, either linear or structural, within the RIFINs may offer better and wider protective base than targeting only a single protein or domain. A recent study has pointed that this is indeed possible as broadly reactive antibodies were generated through insertion of a large DNA fragment between V and DJ segments of antigen binding domains able to recognize RIFINs of different *P. falciparum* isolates (36). Identification and characterization of the cross-protective mechanisms need to be critically pursued.

MSP3 was first identified in 1994 (40), has been a well-known target of naturally acquired immunity, and has been considered to be a promising asexual blood-stage malaria vaccine candidate (41). After the decades of research and development efforts, MSP3 is now under the clinical development as GMZ2, an asexual blood-stage malaria vaccine in combination with another surface antigen, *P. falciparum* glutamate-rich protein (GLURP) (42–44). PfMAAP was recently identified by Aniweh et al. (45). Recently studied for its viability as vaccine candidate, PfMAAP was found localized to the apical region of merozoites, anti-PfMAAP antibodies inhibited merozoite invasion of erythrocytes *in vitro*, and naturally acquired human antibodies to the conserved N- and C- terminal regions of PfMAAP were associated with reduced risk to malaria. Independent analyses and association with protection in this study supports the vaccine candidacy of both MSP3 and PfMAAP, similar to the highly immunogenic erythrocyte surface antigens.

The need for continued development of robust malaria intervention tools including vaccines across different malaria endemicities (46) calls for “flexible” definitions of clinical malaria (47). Moreover, given that malaria can be defined in very diverse ways since clinical presentations vary widely, it is not obvious what aspects are key to supporting accurate monitoring in the face of changing epidemiology (48). We show that three different parasitemia cut-off points yielded almost similar results (**Figure 2A**) suggesting that, for immunological studies, combination of antigens provide an important signature that can be true in different geographical settings. We do acknowledge that there are challenges to attribute fever (or other symptoms) with observed parasitemia (49) and additional studies will be needed to validate or optimize our findings in different settings and in a larger dataset.

We investigated whether a specific combination of antibody responses to the recombinant proteins evaluated could be associated with key malaria severity factors such as age. PCA is useful for exploring multi-dimensional data, mixed infections and complex host-parasite interactions. To this end, and to capture the joint effects of all antibodies in a single analysis in biological conditions, we investigated the relationship between the overall antibody responses by PCA, a factor analysis approach accommodating the fact that protective antibody responses are likely to involve a number of antibodies working together. With the current dataset, the first three principal components accounted for the majority of the outcomes observed (**Figure 3A**). The analyses only showed a significant relationship between first (Dim1) component scores and age; with PfEMP1 domains being the top contributing variables (**Figure 4**; **Table 2**). This is consistent with a recent proposal suggesting that there are ordered acquisition of antibodies targeting PfEMP1 (50). Taken together, these findings suggest that antibodies to multiple proteins may be acquired with age, and continuous exposure is a key factor in protection against clinical disease and protective immunity (2).

Multiple *P. falciparum* protein microarrays have been designed over the past decade to identify malaria vaccine candidate antigens. One of the most important characteristic in this study is the use of WGCFS for recombinant protein production. In contrast to *E. coli*- (5, 51) and mammalian cell-based protein microarray (52) systems which may cause an artefactual change in protein folding (53), WGCFS can express large and complex non-glycosylated proteins in their near-native forms without codon optimization (54). The AlphaScreen immunoscreening platform does not require protein purification and conjugation. Based on these characteristics, therefore, WGCFS/AlphaScreen platform provides a rapid, straightforward tool for screening and identification of parasite antigenic targets as well as protein-protein interactions important for immunity and pathogenesis (55–57), overcoming most challenges attributed to the *E. coli*-based systems (58, 59) resulting to robust evaluations of immunoreactivities across different proteins families and groups.

In conclusion, the large platform of malaria antigens and the analysis approach applied in this study improved our ability to interrelate immunological data with clinical outcomes and highlighted antigens for future work aimed towards developing additional tools for malaria elimination and eradication.

## Data Availability Statement

The original contributions presented in the study are included in the article/**Supplementary Material**. Further inquiries can be directed to the corresponding authors.

## Ethics Statement

The studies involving human participants were reviewed and approved by Institutional Review Committees of Ehime University and Institutional Review Committees of Research Institute for Microbial Diseases, Osaka University, Japan. In Uganda, the studies were reviewed and approved by Lacor Hospital (LHIREC 023/09/13), and Uganda National Council for Science and Technology (HS866, HS1403). Written informed consent to participate in this study was provided by the participants’ legal guardian/next of kin.

## Author Contributions

ET and BK conceived and designed experiments. HN, MM, and IH conducted experiments. ET, BK, and TT analyzed the data. ET, BK, NP, TE, TH, JG, and TT wrote the manuscript. All authors discussed and edited the manuscript.

## Funding

This work was funded in part by JSPS KAKENHI (grant numbers JP20H03481, JP21H02724, JP21KK0138) and in part by Strategic Promotion of International Cooperation to Accelerate Innovation in Africa by MEXT, Japan. BK is an EDCTP Fellow under EDCTP2 programme supported by the European Union grant number TMA2020CDF-3203. The funders had no role in study design, data collection and analysis, decision to publish, or preparation of the manuscript.

## Conflict of Interest

The authors declare that the research was conducted in the absence of any commercial or financial relationships that could be construed as a potential conflict of interest.

## Publisher’s Note

All claims expressed in this article are solely those of the authors and do not necessarily represent those of their affiliated organizations, or those of the publisher, the editors and the reviewers. Any product that may be evaluated in this article, or claim that may be made by its manufacturer, is not guaranteed or endorsed by the publisher.
