# Supplementary material for: Meta-Analysis of Human Antibodies Against Plasmodium falciparum Variable Surface and Merozoite Stage Antigens
Source: Front Immunol. 2022 Jun 9;13:887219. doi: 10.3389/fimmu.2022.887219 (PMC9218060; doi:10.3389/fimmu.2022.887219)
Supplement: Supplementary file 1 [file DataSheet_1.pdf]

Supplementary Figures

Meta-analysis of human antibodies against *Plasmodium falciparum* variable surface and merozoite stage antigens

Eizo Takashima[1‡], Bernard N. Kanoi[2‡], Hikaru Nagaoka[1], Masayuki Morita[1], Ifra Hassan[1], Nirianne M. Q. Palacpac[3], Thomas G. Egwang[4], Toshihiro Horii[3], Jesse Gitaka[2], Takafumi Tsuboi[5*]

[1]*Division of Malaria Research, Proteo-Science Center, Ehime University, Matsuyama, Japan*
[2]*Centre for Research in Infectious Diseases, Directorate of Research and Innovation, Mount Kenya University, Thika, Kenya*
[3]*Department of Malaria Vaccine Development, Research Institute for Microbial Diseases, Osaka University, Suita, Japan*
[4]*Med Biotech Laboratories, Kampala, Uganda*
[5]*Division of Cell-Free Sciences, Proteo-Science Center, Ehime University, Matsuyama, Japan*

Correspondence:
Takafumi Tsuboi, Email: tsuboi.takafumi.mb@ehime-u.ac.jp

[‡]*These authors have contributed equally to this work*

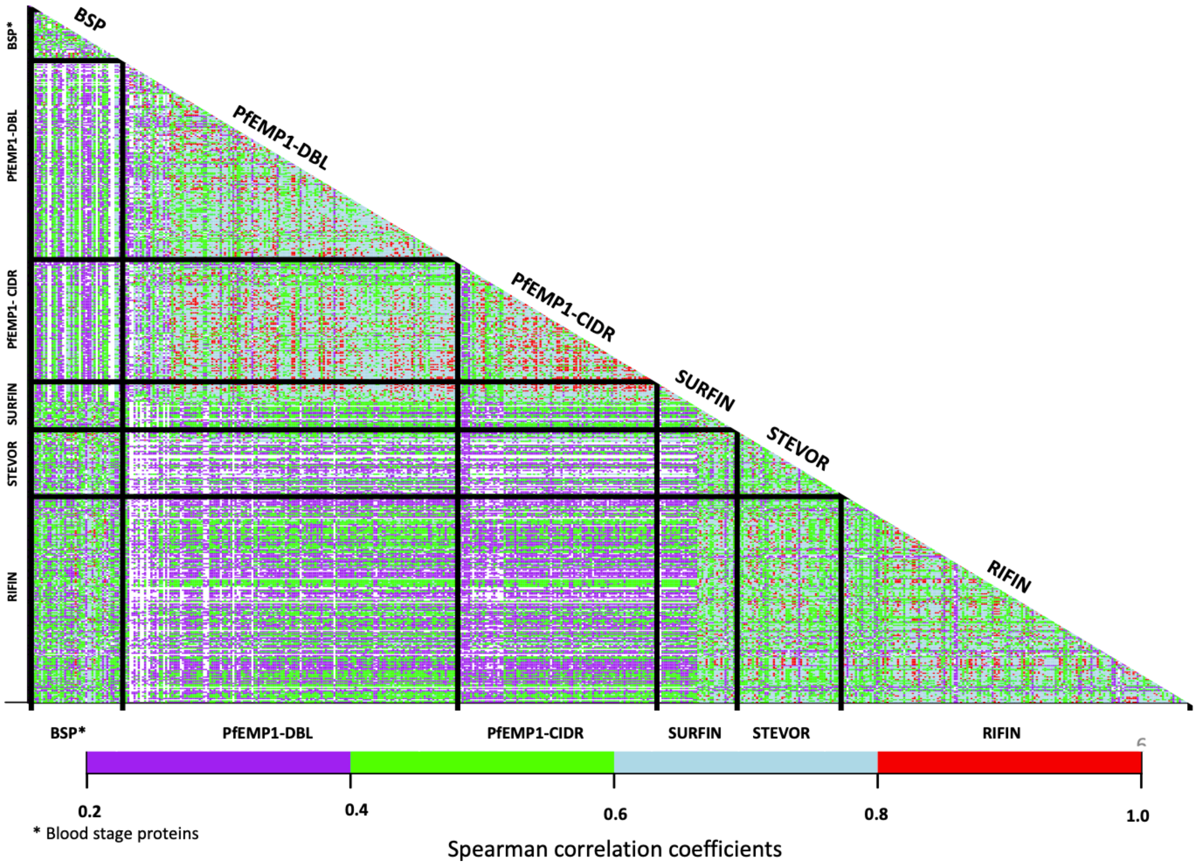

FIG S1: Antibody acquisition correlation matrix. The value score for the Spearman correlation coefficients are shown at the bottom of the matrix by the color scale, with red being 1 correlation and purple being 0.2-0.4 correlation coefficient.

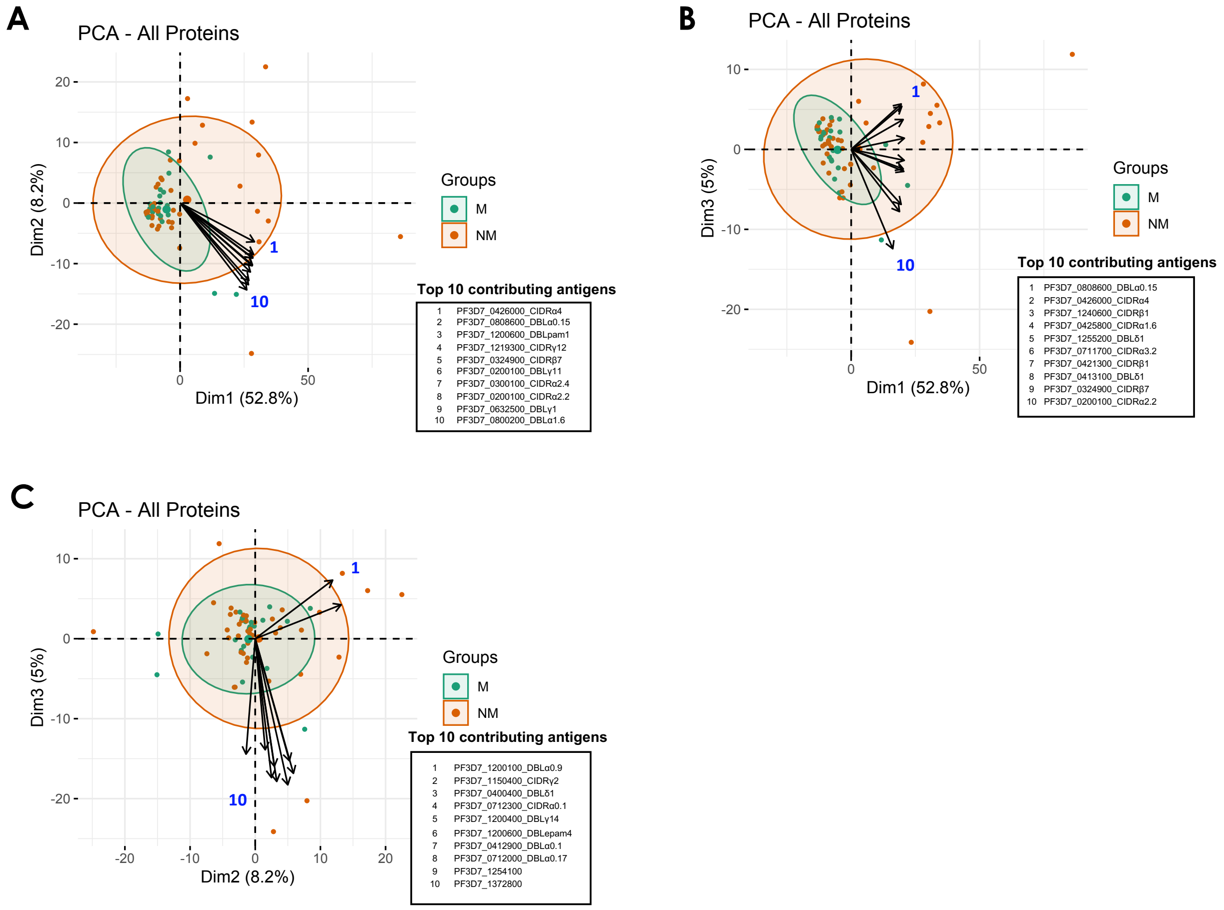

FIG S2: Principal Component Analysis (PCA) biplot showing the distribution of individuals by type of antibody responses to different proteins. Green and red dots represent malaria (M) and no malaria (NM) cases, respectively. Overlapped dots of similar color appear larger. Light green and light red eclipses represent distribution of individuals with and without clinical malaria episodes, respectively. Black arrows indicate the direction of maximum increase and strength (through the length) of the first 10 antigens that contributed to the principal components to the overall distribution. The arrow number #1 represent the antigen with the highest contribution to principal component while #10 represents that in the 10th position in a counted clockwise. These top 10 antigens are shown inset. The contribution of each component to the variable is shown next to the axis label (Dim1, 52.8%; Dim2 8.2%; Dim3 5%). Also presented in Table 2

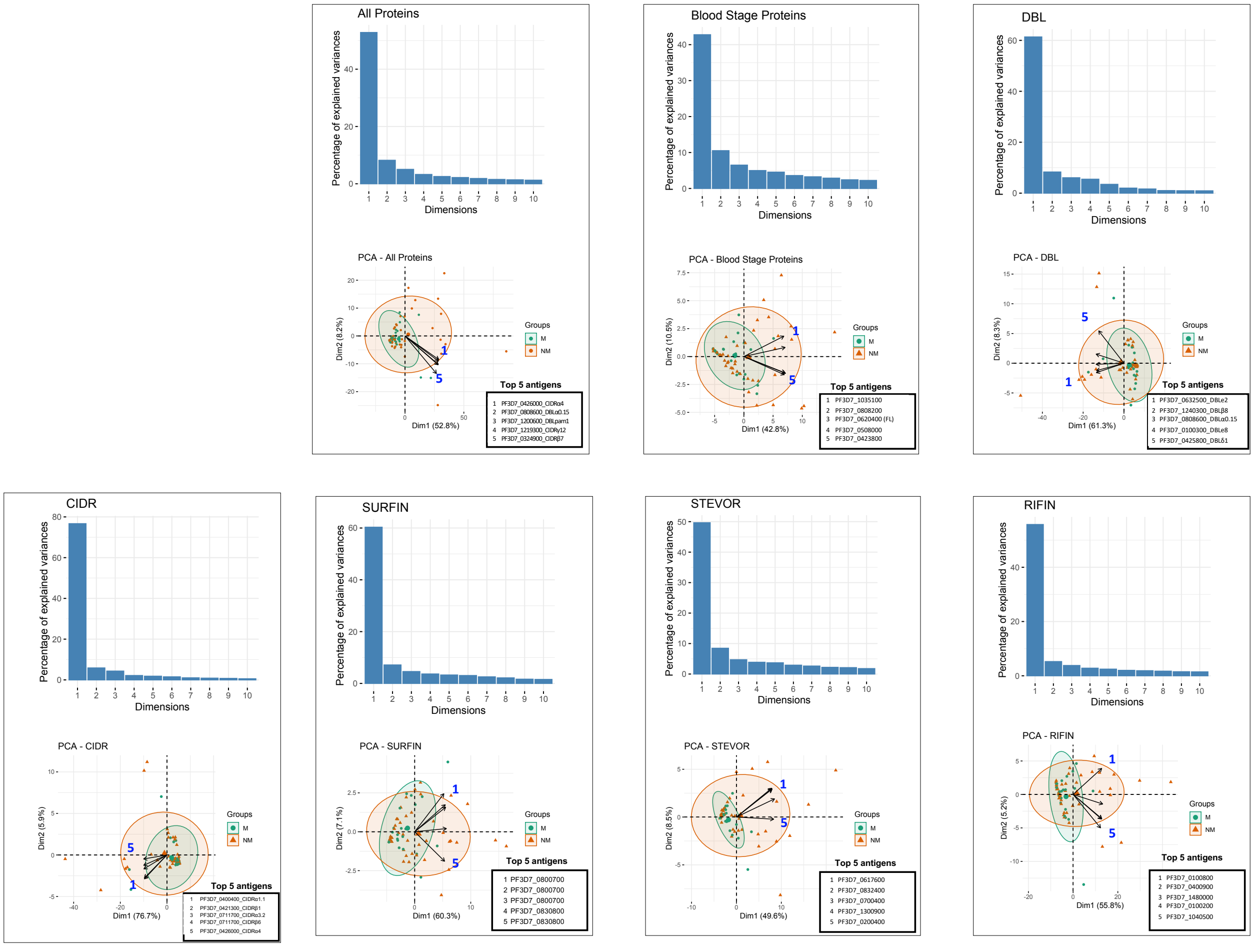

FIG S3: Distribution of Principal components (first 10 dimensions) that explained the highest variance of the antibody responses derived from different protein groups (Upper bar graphs). The plots of the distribution of individuals were shown in the Lower scatter graphs. Green and red dots represent malaria (M) and no malaria (NM) cases, respectively. Overlapped dots of similar color appear larger. Light green and light red eclipses represent distribution of individuals with and without clinical malaria episodes, respectively. Black arrows indicate the direction of maximum increase and strength (through the length) of the first 5 antigens that contributed to the principal components to the overall distribution. The arrow number #1 represent the antigen with the highest contribution to principal component while #5 represents that in the 5th position when counted clockwise. These top 5 antigens are shown inset. The contribution of each component to the variable is shown next to the axis label.
